# Effectiveness and cost-effectiveness of the Pain-at-Work Toolkit for improving work ability in adults with chronic pain: protocol for a cluster randomised controlled trial

**DOI:** 10.1136/bmjopen-2026-123598

**Published:** 2026-09-09

**Authors:** Holly Blake, Wendy J Chaplin, Victoria Abbott-Fleming, Gordon Taylor, Paul McNamee, Melanie Narayanasamy, Angela Thornton, Karen Walker-Bone, Yeliz Prior

**Affiliations:** 1 School of Health Sciences, University of Nottingham, Nottingham, UK; 2 NIHR Biomedical Research Centre: Nottingham, Nottingham University Hospitals NHS Trust, Nottingham, UK; 3 Arthritis UK Pain Centre, University of Nottingham, Nottingham, UK; 4 The British Pain Society, London, UK; 5 College of Medicine and Health, University of Exeter, Exeter, UK; 6 Health Economics Research Unit, Institute of Applied Health Sciences, University of Aberdeen, Aberdeen, UK; 7 Monash Centre for Occupational & Environmental Health, Monash University, Melbourne, Victoria, Australia; 8 Centre for Human Movement and Rehabilitation, School of Health and Society, University of Salford, Salford, UK

**Keywords:** Chronic Pain, Workplace, Randomized Controlled Trial, Digital Technology, Self-Management

## Abstract

**Objectives:**

Chronic pain affects around 28 million adults in the UK and is associated with impaired work ability, reduced productivity and increased sickness absence. Access to work-focused support within healthcare services is limited, and most employers do not routinely provide structured assistance for employees living with chronic pain. The Pain-at-Work Toolkit is a co‑created, web‑based intervention designed to improve work ability, self‑management, and workplace experiences for employees living with chronic pain. A feasibility trial demonstrated strong acceptability, exceptional recruitment and potential improvements in work ability, providing clear justification for progression to a fully powered evaluation. This paper describes the protocol for a definitive cluster randomised controlled trial to evaluate the effectiveness, cost-effectiveness and implementation of the Pain-at-Work Toolkit.

**Methods and analysis:**

This two-arm, open-label cluster-randomised controlled trial will recruit at least 70 organisations (minimum 35 clusters per arm) and at least 685 participants. Organisations will be randomised 1:1 to (a) support-as-usual (SAU) or (b) SAU plus the Pain-at-Work Toolkit, and Pain-at-Work Manager’s Toolkit as an implementation-support component. The primary outcome is work ability at 3 months, measured using the Work Ability Index (three-item version). Secondary outcomes include work self-efficacy, sickness absence, presenteeism, productivity loss, job satisfaction, job stressfulness, turnover intentions, anxiety, depression and health-related quality of life. A mixed-methods process and implementation evaluation will assess fidelity, contextual influences and mechanisms of impact. A health economic evaluation will estimate cost-effectiveness from employer and societal perspectives. Analyses will follow intention-to-treat principles using multilevel modelling.

**Ethics and dissemination:**

Ethical approval was granted by the University of Nottingham Faculty of Medicine and Health Sciences Research Ethics Committee (Ref: FMHS 1200226) and the UK Health Research Authority and Health and Care Wales (IRAS 367449). Findings will be disseminated through peer-reviewed publications, conference presentations, stakeholder reports and public summaries.

**Trial registration number:**

NCT07600892.

**STRENGTHS AND LIMITATIONS OF THIS STUDY**

- Cluster randomisation at organisational level reduces the risk of contamination between intervention and control participants within workplaces.
- Recruitment across public, private and third-sector organisations is expected to improve the applicability of findings across diverse employment settings.
- The study incorporates an embedded mixed-methods process and implementation evaluation using quantitative, qualitative and intervention usage data.
- The open-label design means that participants and researchers cannot be blinded to group allocation.
- Employer-recorded sickness absence data will not be available from all participating organisations, potentially limiting the completeness of some analyses.

## Introduction

 Chronic pain affects approximately 28 million adults in the UK and substantially limits individuals’ capacity to maintain productivity, experience fulfilment at work and remain active participants in the labour market.^1 2^ Despite the high prevalence, access to work-focused support within healthcare services remains limited, and most employers do not routinely provide structured assistance for employees living with chronic pain.^3–6^ These gaps contribute to reduced work ability, increased sickness absence, presenteeism and long-term labour market disengagement.^7^

To address these inequities, the Pain-at-Work Toolkit^8^ was developed (by authors HB and VA-F) as a web-based resource co-created with people living with pain, healthcare professionals and employers. The Toolkit provides educational materials, self-management strategies and guidance on workplace rights and support pathways. It is designed to be flexible, accessible and inclusive, with content tailored to known enablers and barriers to engagement in digital interventions for people with chronic pain. The Toolkit is grounded in a theory of change that posits that increasing employees’ knowledge, confidence and access to support will improve self-management of chronic pain at work, ultimately enhancing work ability, job satisfaction and productivity.

A preliminary feasibility trial conducted in England demonstrated that the Pain-at-Work Toolkit is both acceptable and potentially beneficial for employees experiencing chronic pain.^9^ Recruitment exceeded expectations, with 18 organisations and 380 employees participating—125% and 217% above target, respectively—indicating a clear unmet need for employer-provided support. Employees valued the Toolkit’s content, usability and flexibility, and preliminary findings suggested improvements in perceived work ability at 6 months. Employers also reported that they valued being able to offer the Toolkit to their workforce. Based on feasibility findings, the Toolkit has been updated to ensure relevance across the UK, including content tailored to differences in disability legislation between Great Britain and Northern Ireland.

A definitive evaluation is now required to determine the effectiveness, cost-effectiveness and implementation of the Pain-at-Work Toolkit at scale. This study will address two overarching research questions: (RQ1) What is the effectiveness and cost-effectiveness of the Pain-at-Work Toolkit? and (RQ2) How is the Toolkit delivered, received and used, and how are these processes shaped by contextual factors? The trial design draws on the Medical Research Council (MRC) framework for evaluating complex interventions^10^ and incorporates a built-in process and implementation evaluation to understand mechanisms of impact, contextual influences and strategies for future scale-up and sustainability.

The Pain-at-Work Toolkit was developed through a structured co-creation process involving iterative workshops with people living with chronic pain, employers, occupational health professionals and clinicians.^8 11^ This process ensured that content reflected real-world challenges such as fluctuating symptoms, stigma, disclosure concerns and inconsistent access to workplace adjustments. The development team also incorporated insights from the digital health engagement literature, including the importance of autonomy, personalisation and low-burden design for people managing long-term conditions. The resulting theory of change proposes that improved knowledge, confidence and communication—combined with practical self-management strategies—will enhance work ability and reduce the negative impact of chronic pain on work participation.

The feasibility trial^12 13^ provided important insights that informed the definitive trial design. Engagement analytics showed that most users accessed multiple Toolkit sections, with the ‘Self-management strategies’ and ‘Work capacity, advice and support’ sections those most frequently visited. Participants reported that the Toolkit helped them feel more confident discussing their needs at work and applying pacing strategies. Fidelity assessment indicated that the digital format supported consistent delivery across organisations. These findings informed refinements to content structure, navigation and the timing of engagement reminders, and supported the selection of the 3-month primary endpoint, which showed early responsiveness in feasibility testing.

A cluster randomised controlled trial design is appropriate because the intervention is delivered at the organisational level and individual-level randomisation would risk substantial contamination, with employees in the same workplace sharing information or resources across trial arms. The choice of a 3-month primary endpoint is informed by feasibility findings, which demonstrated early responsiveness in work ability and engagement patterns within this timeframe, and reflects the point at which meaningful short-term changes in self-management, communication and workplace support are expected to emerge following exposure to the Toolkit.

## Methods and analysis

### Study design

**Figure 1:**
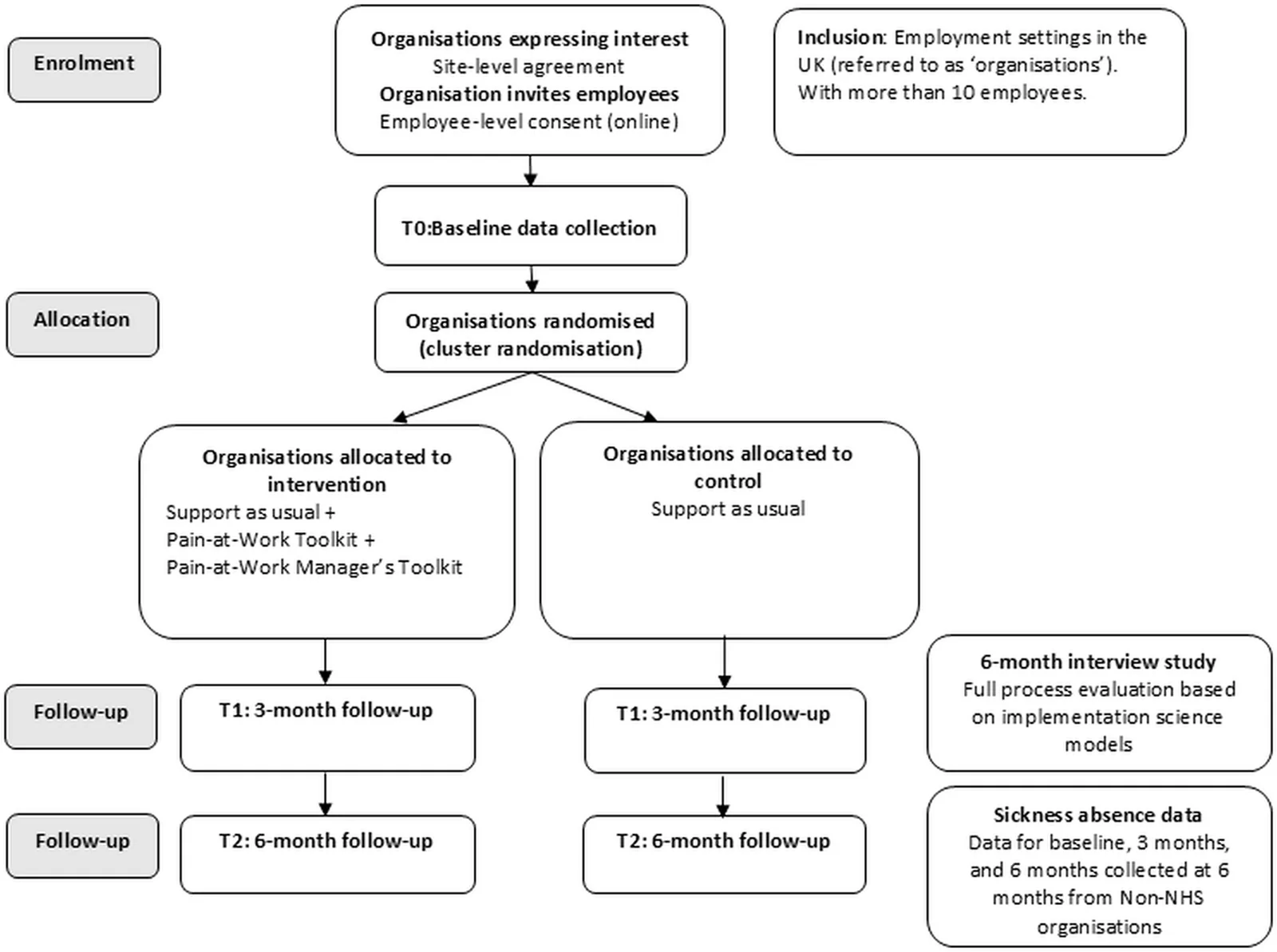
Consolidated Standards of Reporting Trials (CONSORT) participant flow diagram.

This study is a two-arm, open-label cluster randomised controlled trial (cRCT) comparing support-as-usual (SAU) with SAU plus the Pain-at-Work Toolkit, and the Pain-at-Work Manager’s Toolkit. The trial incorporates three work packages: (WP1) effectiveness evaluation, (WP2) health economic evaluation and (WP3) process and implementation evaluation. The design draws on MRC guidelines for evaluating complex interventions.^10^ Reporting conforms to relevant guidelines and frameworks, including Standard Protocol Items: Recommendations for Interventional Trials (SPIRIT)^14^ (online supplemental file 1), Consolidated Standards of Reporting Trials (CONSORT) for cluster trials^15^ (figure 1); Web-based Workforce Health Intervention Development and Evaluation Framework (WWHIDE) for evaluating digital interventions in a workplace setting^16^; Template for Intervention Description and Replication (TIDieR) checklist for intervention description^17^; and relevant frameworks for implementation of digital self-management interventions^18^ and process evaluations in cluster trials.^19^ Intervention evaluation draws on the Technology Acceptance Model^20^ and behaviour change theory.^21^ The study started on 11 May 2026 (date the first participant was enrolled) and the estimated study completion date (completion of participant data collection) is 30 September 2028.

### Setting

The study will be conducted in UK employment settings across public, private and third sectors. Organisations will vary in size (small: 10–49 workers; medium: 50–249 workers; large: >250 workers). Worksites within larger organisations may be treated as independent clusters if geographically separated by ≥1 km, consistent with prior workplace cRCTs.^22^

### Participants

#### Eligibility criteria

Participating organisations must employ at least 10 staff, be based in the UK, and be able to support study procedures and provide organisational-level data. Participating organisations must employ at least ten staff to ensure a minimum viable cluster size for recruitment and to reflect organisational capacity to support study procedures and internal dissemination. Employees are eligible if they are aged 18 years or older, in paid employment (including full-time, part-time, permanent, contracted, subcontracted or gig work) and self-identify as experiencing chronic pain that affects their ability to undertake or enjoy productive work. Eligible participants must be able to provide informed consent and comprehend written English, as this is required for both the consent process and use of the Toolkit. Unpaid employees are excluded due to differing legal rights and employment relationships, and individuals who are unable to comprehend written English are excluded because the current version of the Toolkit is available only in English.

### Recruitment and consent

Feasibility work demonstrated that organisations were motivated to participate due to increasing awareness of workforce well-being, rising sickness absence related to musculoskeletal and pain conditions, and a desire for practical tools to support staff. Employees reported that the study invitation felt relevant and timely, particularly where workplace support had previously been limited. These insights informed the recruitment strategy, including the use of clear, accessible study information and multiple communication channels within organisations.

### Organisation recruitment

As with the feasibility study, organisations will be identified through professional networks, employer associations and advisory groups. Interested organisations will receive study information and undergo eligibility screening. A designated employer representative (eg, HR manager, occupational health lead, line manager or company owner) will provide organisational agreement.

### Employee recruitment

Participating organisations will disseminate study information to all employees using usual communication channels. Employees will self-identify eligibility and provide online informed consent (online supplemental files 2 and 3, for NHS and non-NHS employees, respectively) before completing baseline measures. Employees who do not routinely use email or online systems may complete consent and baseline measures via telephone with the research team.

Recruitment will continue until at least 70 organisations (minimum 35 clusters per arm) and at least 685 participants are enrolled. Recruitment feasibility is supported by the prior feasibility trial, which exceeded targets (18 organisations and 380 employees).^9^

### Randomisation

Participating organisations (clusters) will be randomised after baseline assessment using a 1:1 allocation ratio, stratified by organisation size. The trial statistician will generate the allocation sequence and provide the allocation information to the study team. The allocation sequence will be generated independently by the trial statistician using computer-generated randomisation. Allocation will be concealed until baseline data collection is complete for each matched pair of organisations. Where feasible, statisticians undertaking the primary analysis will remain blinded to group allocation until the analysis plan is finalised. Due to the nature of the intervention, blinding of participants and researchers is infeasible.

### Intervention

#### Pain-at-Work Toolkit (employee version)

The Pain-at-Work Toolkit^8 23^ is a web-based resource offering evidence-based advice on chronic pain, disability rights, work capacity, self-management strategies (eg, pacing, self-care) and signposting to additional support. Content was co-created with people living with pain, healthcare professionals and employers. The Toolkit is accessible on any device, at any time and can be used at the user’s preferred pace. Content is available in two versions: Great Britain (England, Scotland, Wales) and Northern Ireland, reflecting differences in disability legislation and available employee support.

The Toolkit comprises five sections:

1. Understanding chronic or persistent pain.
2. Pain and disability.
3. Work capacity, advice and support.
4. Self-management strategies.
5. Resources.

Each of the five Toolkit sections maps to specific mechanisms of impact. ‘Understanding chronic or persistent pain’ provides psychoeducation to reduce fear and misconceptions. ‘Pain and disability’ supports informed decision-making about disclosure and rights. ‘Work capacity, advice and support’ guides users through conversations with managers and occupational health. ‘Self-management strategies’ offers pacing, activity regulation, and self-care tools. ‘Resources’ consolidates signposting to trusted organisations and services. The Toolkit was updated following the feasibility trial to improve clarity, navigation and accessibility, and to ensure legal accuracy across UK nations.

The intervention is grounded in a theory of change and draws on persuasive systems design,^24^ the Technology Acceptance Model^20^ and behaviour change theory.^21^ Fidelity and engagement were demonstrated in the feasibility trial.^9^

The Toolkit was developed using a structured co-creation methodology,^8^ including focus groups, usability testing and iterative refinement cycles with employees living with chronic pain. Participants emphasised the need for flexible, non-judgemental content that validated lived experience and provided actionable strategies. The final Toolkit incorporates behaviour change techniques such as goal-setting, self-monitoring, problem-solving and environmental restructuring. Persuasive systems design elements include tunnelling, tailoring, reminders and credibility features to enhance engagement and perceived usefulness.^13^ Participants may use the Toolkit as often as they wish, and engagement data from the feasibility study indicated that most users accessed the resource multiple times, with typical use involving two to four logins. Users made repeated visits to key sections such as self-management strategies and work-related advice, and shared resources with managers and colleagues.

### Manager’s Toolkit

The Manager’s Toolkit^25^ is a parallel resource designed to help line managers support employees living with chronic pain. Although the primary intervention is the Pain-at-Work Toolkit for employees, this manager-facing toolkit acts as an essential implementation support component. Developed in response to employer feedback during feasibility testing, the Manager’s Toolkit addresses a commonly reported challenge: managers often feel uncertain about how to effectively support staff experiencing chronic pain. Organisations involved in testing strongly endorsed the idea of providing a dedicated resource for managers. The content aligns closely with the employee Toolkit and will be distributed to all managers within intervention organisations. It offers practical guidance on reasonable adjustments, effective communication strategies and fostering supportive team cultures. By equipping managers with greater confidence and clarity, the Manager’s Toolkit is expected to strengthen organisational readiness and enhance the overall implementation of the intervention.

### Engagement support

To maximise engagement, text message reminders will be sent throughout the intervention period. Messages were developed using the Behaviour Change Wheel and COM-B.^26^ In the feasibility trial, 97% of participants opted in to receive reminders. A ‘Frequently Asked Questions’ section will be available on the trial website to address common queries. Weekly text message reminders were refined through user testing to ensure they were supportive, non-intrusive and aligned with behaviour change principles. Messages include prompts to revisit Toolkit sections, encouragement to apply strategies at work and reassurance about the legitimacy of chronic pain experiences. Engagement data from the feasibility trial showed that reminders were a key driver of sustained use.

### Control condition: SAU

SAU may include occupational health services, counselling, line manager support or signposting to educational resources. The nature of SAU varies across organisations. Employer-reported SAU data will be collected at baseline.

### Outcomes

The primary outcome is work ability at 3 months, measured using the three-item version of the Work Ability Index (WAI).^27^ Work ability is the primary outcome because it captures the core clinical impact of chronic pain on functioning at work and is directly influenced by the behavioural targets of the intervention, such as self-management, communication and use of adjustments. The brief three-item WAI is a validated, sensitive measure that showed improvement in the feasibility trial, making it an appropriate and responsive indicator of change. The items are: (a) 0–10 rating: compared with lifetime best, current work ability; (b) 1–5 rating: current work ability with respect to the physical demands of your work; (c) 1–5 rating: current work ability with respect to the mental demands of your work. The 3-month endpoint was selected to capture early change in work ability following intervention exposure while minimising attrition associated with longer follow-up.

Secondary outcomes span four domains. Work-related outcomes include the Work Productivity and Activity Impairment Questionnaire: General Health,^28^ the Work Self-Efficacy Scale,^29^ the Occupational Self-Efficacy Scale,^30^ subscales of the Demand Control Support Questionnaire^31^ and single-item measures of job satisfaction,^32^ job stressfulness^33^ and turnover intentions^34^; as used by Blake and colleagues.^13^

Psychological and health-related quality of life outcomes include the Generalised Anxiety Disorder scale,^35^ the Patient Health Questionnaire^36^ and the EuroQol 5-Dimension 5-Level instrument (EQ-5D-5L).^37^

Health economic outcomes include healthcare resource use (adapted from validated instruments^38^) and employer-reported sickness absence where available.

Finally, technology acceptance and engagement outcomes include measures mapped to the Technology Acceptance Model (perceived usefulness, ease of use, attitudes and behavioural intention) and Toolkit usage analytics such as logins, time spent and sections accessed.

Employer and participant-reported outcome measures are shown in online supplemental file 4online supplemental file 4 ^39–42^.

### Data collection

Data will be collected at three time points: baseline (T0), 3 months (T1) and 6 months (T2). Participant-reported outcome measures will be administered via online surveys at each time point. Employer-reported sickness absence data will be collected at T2 where available, except for NHS Trusts, from which sickness absence data will not be requested. Sickness absence data are not collected at NHS PIC sites (Participant Identification Centres; their role in the research is to identify participants for the research and not to conduct research themselves) because these organisations cannot provide individual-level absence records in a form compatible with the study’s data-sharing and governance requirements.

### Safety

The methods used in the trial are online surveys and interviews. The Toolkits are informational, requiring viewers to watch short videos and read text. There is nothing prescriptive; participants are free to choose how they use the information. We will capture any exacerbation of pain symptoms and monitor any spontaneously reported adverse events during the trial.

### Sample size

A total sample of 685 employees across at least 70 clusters from across the UK (35 per arm) provides 80% power to detect a 0.6 point difference in WAI (SD 2.0; intracluster correlation coefficient (ICC) 0.02), allowing for 60% attrition and with an expected cluster size of 10 per cluster. This calculation is justified based on estimates from our feasibility trial and an ICC of 0.02 is consistent with values commonly reported in workplace and organisational health research, where ICCs for work-related and psychosocial outcomes typically fall between 0.01 and 0.03 (eg, ^43–45^). Cluster randomisation at the organisational level was selected to minimise contamination arising from the sharing of Toolkit materials or informal peer-to-peer diffusion within workplaces.

### Statistical analysis

Analyses will follow intention-to-treat principles. The primary analysis will compare WAI scores between groups across time using a multilevel mixed-effects model, incorporating all available time points (baseline, 3 months and 6 months). The model will include fixed effects for treatment group, time and group-by-time interaction, and will adjust for baseline WAI and clustering. The primary comparison of interest will be the adjusted between-group difference at 3 months. Secondary analyses will estimate between-group differences at 6 months using the same model framework. Secondary outcomes will be analysed using similar repeated-measures mixed-effects models, including all time points. Responsiveness statistics (standardised response mean and effect size) will be calculated for each patient-reported outcome measure. The amount and distribution of missing data will be examined to determine the type of missing data and appropriate methods to impute will be assessed according to the Statistical Analysis Plan that will be agreed with the Trial Steering Group and Trial Management Group.

### Health economic evaluation

A cost-utility analysis will be conducted from a societal perspective. The economic evaluation will include direct employer costs (eg, sickness absence, presenteeism-related productivity loss and any costs associated with implementing the Toolkit) and broader societal costs such as healthcare utilisation, informal care and reduced productivity outside work. The decision-analytic model will be structured to reflect transitions between states of work ability and productivity over time, informed by trial data and published literature. Sensitivity analyses will explore uncertainty in key parameters, including intervention uptake, engagement levels and variability in organisational support. Differences in total costs and quality-adjusted life years (QALYs) between groups will be estimated using generalised linear models, adjusting for baseline covariates. Uncertainty will be assessed using non-parametric bootstrapping, and cost-effectiveness acceptability curves will be generated. A decision-analytic model will extrapolate costs and QALYs beyond the trial period (5–10 years). Reporting will follow Consolidated Health Economic Evaluation Reporting Standards (CHEERS) guidelines.^46^

### Process and implementation evaluation

A mixed-methods process and implementation evaluation will be conducted to examine how the intervention is delivered, received and enacted across organisational contexts. The process evaluation is informed by implementation science frameworks^47^ including constructs relating to organisational readiness, leadership engagement and compatibility with existing workplace practices. Interview participants will be purposively sampled to ensure variation in organisation size, sector, managerial role and level of Pain-at-Work Toolkit engagement. Interviews will explore how organisational culture, workload pressures and managerial attitudes shape engagement with the Toolkit. Mechanisms of impact will be examined by linking qualitative insights with quantitative measures of technology acceptance, usage patterns and changes in work-related outcomes. This evaluation will identify strategies to support future scale-up, including integration with occupational health pathways and alignment with employer well-being initiatives.

The evaluation will assess fidelity of the Pain-at-Work Toolkit through indicators of reach, exposure, engagement and enactment using usage analytics and participant reports. It will explore contextual influences such as organisational culture, policies and available resources; and identify mechanisms of impact, as well as barriers and facilitators to implementation. It will also generate insights into strategies for future scale-up and sustainability.

Data sources will include Pain-at-Work Toolkit usage analytics, technology acceptance measures, semi-structured interviews with up to 60 employees, managers and wider stakeholders, and organisational data on SAU. Additionally, approximately 30 interviews with managers will explore their views on the acceptability and usefulness of the Pain-at-Work Manager’s Toolkit. Qualitative data will be analysed using reflexive thematic analysis,^48^ with themes mapped to relevant constructs from the Consolidated Framework for Implementation Research (CFIR) (eg, inner setting, leadership, individual characteristics)^49^ and RE-AIM (eg, Reach, Effectiveness, Adoption, Implementation and Maintenance)^50^ to interpret findings through an implementation science lens. This will enable us to identify barriers and facilitators to implementation, explore how fidelity relates to outcomes and inform future scale-up and sustainability.

### Patient and public involvement

People with lived experience of chronic pain were involved throughout the development of the Pain-at-Work Toolkit and the design of this study.^11^ Patient and public involvement (PPI) contributors participated in co-creation workshops which shaped Toolkit content, incorporated usability testing to refine structure and navigation, and informed trial recruitment strategies and engagement materials. They also contributed to interpreting feasibility findings and identifying priorities for refinement ahead of the definitive trial. PPI contributors will continue to be involved in promotion of recruitment materials, interpreting study results, co-authoring academic outputs and co-developing accessible dissemination materials including public summaries. No PPI contributors will be involved in data analysis.

### Trial management

The Trial will be managed by the University of Nottingham, supported by the Trial Management Group. The Trial Management Group is composed of all co-investigators (including PPI, statistical, and health economic representatives) and will meet monthly to oversee day-to-day management of the study and to address any emergent issues. The appointed trial researchers will meet the Chief Investigator monthly to discuss project oversight and operational matters.

A Trial Steering Group, including four independent members, will meet every 6 months to review recruitment, data collection and safety data. The Trial Management Group will submit updates on recruitment, data collection and safety to the Study Steering Group every 6 months. Both the Trial Management and Steering groups are independent of the funder. The Sponsor and the Funder reserve the right to discontinue the trial at any time for failure to meet expected enrolment goals, for safety or for any other administrative reasons. The Sponsor shall take advice from the Trial Steering Committee and Data Monitoring Committee as appropriate in making this decision.

A Trial Advisory Group will meet every 4 months. It will provide recommendations and will respond to situations as they arise (eg, in the instance of low recruitment) and provide guidance on alignment with the broader context in work and health that may influence the interpretation of the study findings (eg, policy or practice advances). It will comprise policymakers, researchers, business advisors, funders and people with lived experience of chronic pain.

## Discussion

This definitive trial will address an important evidence gap regarding scalable workplace support for employees living with chronic pain. Chronic pain is a major and growing contributor to reduced work ability, sickness absence, presenteeism and premature labour market exit in the UK, with substantial consequences for individuals, employers and the wider economy.^7^ Although chronic pain affects a significant proportion of the working-age population,^2^ provision of work-focused support through healthcare services remains limited and fragmented.^1^ At the same time, employers are increasingly expected to support employees with long-term conditions but frequently lack structured, evidence-based resources to do so, particularly for conditions such as chronic pain that are variable, often invisible and poorly accommodated by standard workplace adjustments.^8 48^ This disconnect between need, responsibility and available support represents a persistent and well-recognised gap in occupational health and vocational rehabilitation provision.

The Pain at Work Toolkit was developed to address this gap through a co-created, evidence-based and accessible digital intervention designed specifically to support employees in managing chronic pain at work. While digital health interventions are increasingly promoted as scalable solutions, there remains a lack of robust definitive trial evidence demonstrating their effectiveness, cost-effectiveness, implementation feasibility and sustainability within real-world organisational settings, particularly for chronic pain. As a result, employers, policymakers and health services currently lack the evidence required to justify investment in or large-scale adoption of such interventions.

This definitive trial is therefore both timely and necessary. Building on strong feasibility findings, it aims to provide evidence on the effectiveness of a scalable workplace intervention for chronic pain, while also addressing critical unanswered questions about how, for whom and under what circumstances the intervention works. The trial is explicitly designed not only to assess outcomes but also to elucidate underlying mechanisms of impact, contextual influences on delivery and engagement, and the implementation strategies required for sustainable scale-up. The inclusion of a mixed-methods process and implementation evaluation is a key strength, enabling a nuanced understanding of fidelity, acceptability, engagement and contextual variation across diverse organisational contexts. This trial will help close an important evidence gap and support the development of more effective, equitable and sustainable approaches to supporting people with chronic pain to remain in, and thrive at, work.

The study has several strengths. It is the first large-scale UK trial to evaluate a digital workplace intervention specifically designed for employees with chronic pain. Cluster randomisation minimises contamination and reflects real-world implementation conditions. Recruitment across diverse sectors enhances generalisability and the co-created nature of the intervention ensures relevance to end-users. The integration of a health economic evaluation will provide important insights into value for money for employers and society.

The co-creation approach underpinning the Toolkit is a key strength, ensuring that the intervention reflects the lived realities of employees with chronic pain and the practical constraints faced by employers. The digital format supports scalability and consistency of delivery, addressing longstanding inequities in access to work-focused pain support. By integrating behavioural, organisational and psychosocial components, the Toolkit represents a multi-component approach to improving work participation for people with chronic pain. The inclusion of a comprehensive process evaluation will enable nuanced interpretation of trial outcomes and provide actionable insights for implementation in diverse workplace settings. Including organisations from multiple sectors should enhance transferability, although contextual differences between workplaces may influence intervention uptake and impact.

However, some limitations should be acknowledged. Organisations volunteering to participate may differ from those that do not, potentially introducing selection bias. The intervention is delivered only in English (at this time), which may limit accessibility for some workers. Additionally, employer-reported sickness absence data may not be available from all organisations (eg, NHS Trusts), which may limit completeness of economic analyses. Future implementation is also likely to depend on organisational readiness, managerial engagement, competing operational pressures and digital accessibility.

Despite these limitations, the trial is designed to generate high-quality evidence to inform policy, practice and future implementation. If shown to be effective and cost-effective, the Pain-at-Work Toolkit has the potential to reduce inequalities in workplace support for people with chronic pain, improve work participation and reduce the societal and economic burden associated with chronic pain.

## Ethics and dissemination

Ethical approval for this study was granted by the University of Nottingham Faculty of Medicine and Health Sciences Research Ethics Committee (Ref: FMHS 120 0226) and the UK Health Research Authority and Health and Care Wales (IRAS 367449). The trial is sponsored by the University of Nottingham (reference number 26011; sponsor@nottingham.ac.uk). The sponsor reviewed and approved the study protocol and documents, suggesting a few minor amendments. The sponsor will not have input into data collection, management, analyses or interpretation of data or publication. All participants will provide informed consent prior to taking part. Should a protocol amendment require REC approval, the changes will not be instituted until the amendment and the revised informed consent forms and participant information sheets (if appropriate) have been reviewed and received approval/favourable opinion from the REC and R&D departments. A protocol amendment intended to eliminate an apparent immediate hazard to participants may be implemented immediately, provided that the REC are notified as soon as possible and approval is requested. Minor protocol amendments only for logistical or administrative changes may be implemented immediately, and the REC will be informed.

Data will be managed in accordance with the General Data Protection Regulation and institutional data protection policies. Data will be stored securely for a minimum of 7 years following study completion, in accordance with institutional research data management policies and UK regulatory requirements. Each participant will be assigned a trial identity code number, allocated after consent, for use on Case Report Forms, questionnaires and other trial documents and the electronic database. Their personal data will be secured until all timepoints have been linked to each participant, then the data will be anonymised and all personal data will be deleted.

Study findings will be disseminated through peer-reviewed journal publications, national and international conference presentations, and stakeholder reports prepared for funders, employers, policymakers and professional bodies. Public summaries will be co-developed with patient and public involvement contributors to support wider accessibility and engagement. In line with institutional policies, data and Data Collection Forms from the trial will be made available on reasonable request following study completion. If the Pain-at-Work Toolkit is found to be effective, findings could inform employer well-being strategies, occupational health pathways and policy approaches to supporting work participation among people living with chronic pain.

## Supplementary material

10.1136/bmjopen-2026-123598 online supplemental file 1

10.1136/bmjopen-2026-123598 online supplemental file 2

10.1136/bmjopen-2026-123598 online supplemental file 3

10.1136/bmjopen-2026-123598 online supplemental file 4
