# Supplementary material for: Effectiveness and cost-effectiveness of the Pain-at-Work Toolkit for improving work ability in adults with chronic pain: protocol for a cluster randomised controlled trial
Source: BMJ Open. 2026 Sep 9;16(9):e123598. doi: 10.1136/bmjopen-2026-123598 (PMC13560969; doi:10.1136/bmjopen-2026-123598)
Supplement: Supplementary file 1 [file bmjopen-16-9-s002.pdf]

Faculty of Medicine & Health Sciences
School of Health Sciences
B Floor, Medical School
Queen's Medical Centre
Nottingham
NG7 2HA

PARTICIPANT ONLINE CONSENT FORM
(Draft Version 1.1 / Final version 1.0: 17 April 2026)

Title of Study: Pain-at-Work Toolkit for NHS Employees with Chronic Pain

IRAS Project ID: 367449

Chief Investigator: Professor Holly Blake
Project Researcher: Dr Wendy Chaplin

Name of Participant:

1. I confirm that I have read and understand the information sheet version number 1.0 dated 17.04.2026 for the above study and have had the opportunity to ask questions. ☐
2. I understand that my participation is voluntary and that I am free to withdraw at any time, without giving any reason, and without disadvantage. ☐
3. I understand that relevant sections of my data collected in the study may be looked at by authorised individuals from the University of Nottingham, the research group and regulatory authorities where it is relevant to my taking part in this study. I give permission for these individuals to have access to these records and to collect, store, analyse and publish information obtained from my participation in this study. I understand that my personal details will be kept confidential. ☐
4. I understand that I may be invited for an interview which is optional. Any interviews with myself, may be video- or audio-recorded and that anonymous direct quotes from the interview may be used in the study reports. Yes ☐ No ☐
5. I understand that information about my participation recorded during the study will be made anonymous before it is stored. It will be uploaded into a secure database on a computer and kept in a secure place. Data will be kept for 7 years after the study ends, then deleted. ☐
6. I understand that the information collected about me may be used to support other research in the future, and may be shared anonymously with other researchers. ☐
7. I understand that what I say in survey responses and during any interviews will be kept confidential unless they reveal something of concern that may put themselves or someone else at any risk. It will then be necessary to report this to the appropriate persons. ☐
8. I understand that my email address and first name, mobile number (optional) will be stored in the SmartSurvey platform to allow surveys and SMS texts to be sent to me. All data will be handled by University of Nottingham research staff and I can request to opt out of SMS texts. ☐
9. I would like to receive a summary of the results at the end of the study (optional) Yes ☐ No ☐
10. I agree to take part in the above study. ☐

First name	Middle initial (or -)	Family name
____________	____	____________

Faculty of Medicine & Health Sciences
School of Health Sciences
B Floor, Medical School
Queen's Medical Centre
Nottingham
NG7 2HA

NHS Trust

Year of birth

This document will automatically be date and timestamped.
