# Supplementary material for: Effectiveness and cost-effectiveness of the Pain-at-Work Toolkit for improving work ability in adults with chronic pain: protocol for a cluster randomised controlled trial
Source: BMJ Open. 2026 Sep 9;16(9):e123598. doi: 10.1136/bmjopen-2026-123598 (PMC13560969; doi:10.1136/bmjopen-2026-123598)
Supplement: Supplementary file 3 [file bmjopen-2026-123598.reviewer_comments.pdf]

PEER REVIEW HISTORY

BMJ Open publishes all reviews undertaken for accepted manuscripts. Reviewers are asked to complete a checklist review form and are provided with free text boxes to elaborate on their assessment. These free text comments are reproduced below.

ARTICLE DETAILS

Title (Provisional)

Effectiveness and cost-effectiveness of the Pain-at-Work Toolkit for improving work ability in adults with chronic pain: protocol for a cluster randomised controlled trial

Authors

Blake, Holly; Chaplin, Wendy J; Abbott-Fleming, Victoria; Taylor, Gordon; McNamee, Paul; Narayanasamy, Melanie; Thornton, Angela; Walker-Bone, Karen; Prior, Yeliz

---

This article was not externally reviewed at BMJ Open. Protocols that have been independently assessed prior to submission to BMJ Open are usually fast-tracked to publication on the grounds that further substantial changes will not be possible. This independent assessment will usually be external, independent review for both a substantial grant award from a non-commercial or government funder and ethics approval.
