# Supplementary material for: Effectiveness and cost-effectiveness of the Pain-at-Work Toolkit for improving work ability in adults with chronic pain: protocol for a cluster randomised controlled trial
Source: BMJ Open. 2026 Sep 9;16(9):e123598. doi: 10.1136/bmjopen-2026-123598 (PMC13560969; doi:10.1136/bmjopen-2026-123598)
Supplement: Supplementary file 5 [file bmjopen-16-9-s004.docx]

Supplementary file 4. Employer and participant-reported outcome measures

| **Data collection** | **Measurement Methods** | **Details** | **T0** | **T1** | **T2** |
| --- | --- | --- | --- | --- | --- |
|  | |  |  |  |  |
| **Employer-reported outcomes** | |  |  |  |  |
|  | | Data collected by online survey/email (i) and secure data transfer (ii). |  |  |  |
| i. **Organisation (at recruitment)** | |  |  |  |  |
|  | Online survey/email | Employment setting characteristics: Sector, type, and size; number of staff; role of the ‘gatekeeper’ employee representative; organisation's views on workplace culture; description of SAU; existing provisions to support staff with long-term health conditions. | ✓ | ✓ | ✓ |
|  |  |  |  |  |  |
| ii. **Sickness absence rates** |  |  |  |  |  |
|  | Employer records | About participating employees (with consent). | ✓ | ✓ | ✓ |
|  |  |  |  |  |  |
| **Participant-reported outcome measures (PROMs)** | | . |  |  |  |
|  | | Data collected by online survey. |  |  |  |
| i. **Socio-demographic** | |  |  |  |  |
|  | Age | Continuous measure (≥18 years). | ✓ | ✓ | ✓ |
|  | Sex at birth | What sex were you assigned at birth? | ✓ | ✓ | ✓ |
|  | Gender identity | Female/male/prefer not to state/self-definition (FT). | ✓ | ✓ | ✓ |
|  | Ethnic Group | White/ Black- Caribbean/ Black-African/ Black-other/ Indian/ Pakistani/ Bangladeshi/ Chinese/ Other (FT) | ✓ | ✓ | ✓ |
|  | Living arrangements status | Alone/ with family or significant others/ other. | ✓ | ✓ | ✓ |
|  | School leaving age | Continuous variable. | ✓ | ✓ | ✓ |
|  | Direct transfer from school to full-time education/ university | Yes/ no. | ✓ | ✓ | ✓ |
|  | Educational attainment | 7 classes: GCSEs, A levels, Vocational, University degree, Higher National Diplomas, Professional, None. | ✓ | ✓ | ✓ |
|  | Household income yearly total (£) | 14 classes: <9,999; 10,000 – 19,999; 20,000 – 29,999; 30,000- 39,999; 40,000 – 49,999; 50,000 – 59,999; 60,000 – 69,999; 70, 000 - 99,999 ; 100,000 – 149, 999 ; 150,000 - 199,999; 200,000 – 250, 000; 250,000 – 499, 999; >500, 000; Prefer not state. | ✓ | ✓ | ✓ |
|  | Do you have children/dependents? | Yes/ No / Prefer not to state | ✓ | ✓ | ✓ |
|  | Do you consider yourself a carer? | Yes/ No / Prefer not to state | ✓ | ✓ | ✓ |
|  | Do you consider yourself to have a disability? | Yes/ No / Prefer not to state | ✓ | ✓ | ✓ |
| ii. **Health** |  |  |  |  |  |
| Pain (present) | Numerical rating scale [38] | Continuous scale 0-10. ‘On a scale from 0 to 10, where 0 is no pain, and 10 is the worst pain you’ve experienced, at this moment, what number represents your overall pain level?’ | ✓ | ✓ | ✓ |
| Pain duration |  | Years and months. | ✓ |  |  |
| Comorbidities |  | 9 morbidities: present/ absent/ not sure.  Morbidities: high blood pressure; heart problems; diabetes; kidney disease; stroke or TIA; arthritis; asthma or lung conditions; anxiety or depression; liver or stomach problems; other (FT). | ✓ |  |  |
|  | Do you experience chronic or persistent pain in the following (Select all that apply) | Muscles, joints or bones (e.g. back, neck, hip, knee, shoulder, hands, feet) | ✓ |  |  |
|  |  | Head or face (e.g. headaches, migraine, facial pain, jaw pain) | ✓ |  |  |
|  |  | Nerves (e.g. sciatica, nerve pain, burning or shooting pain) | ✓ |  |  |
|  |  | Abdomen or pelvis (e.g. IBS, period pain, pelvic pain) | ✓ |  |  |
|  |  | Widespread pain affecting many areas (e.g. fibromyalgia) | ✓ |  |  |
|  |  | Other chronic or persistent pain (please specify) (free Text)  If yes, tell us___________ | ✓ |  |  |
|  |  |  |  |  |  |
| Height and weight | Metric or imperial | Calculation of Body Mass Index [Weight (kg) / height^2^ (m)]. | ✓ | ✓ | ✓ |
|  |  |  |  |  |  |
| iii. **Employment characteristics of the employee** | |  |  |  |  |
|  | | Sector, size and type of employing organisation, assessment of their perception of organisation culture, and which SAU services or support they have accessed via their employer (SAU may consist of (but is not limited to) any combination of the following: occupational health, counselling, line manager support, signposting to education about factors that may have positive or negative effects on chronic pain). | ✓ | ✓ | ✓ |
|  | |  |  |  |  |
| Current employment status. |  |  |  |  |  |
|  | Have you disclosed your pain condition and/or health condition to your employer? | Yes/ No / Prefer not to state | ✓ | ✓ | ✓ |
|  | Work Transitions Index (WTI) (part 1) [39] | 12 classes: Full-time/part-time/ leave of absence/ short-term sickness/ long-term sickness/ unemployed/ not employed/ retired/ early retirement on grounds of ill health/ homemaker/ other (FT). Sick leave: start date. | ✓ | ✓ | ✓ |
|  | Job skill level 1-4 (UK standard Occupational Classification | Job title, industry, and number of paid hours: 1/52. Main and up to 2 other jobs. | ✓ | ✓ | ✓ |
|  | Total hours and work pattern. | Average 1/52. Contracted and actual. Number of working days 1/7. | ✓ | ✓ | ✓ |
|  | Sole income status | Yes/ no. | ✓ | ✓ | ✓ |
|  | Job sector classification [40] | Standard Occupational Classification: 9 major classes | ✓ | ✓ | ✓ |
|  | Self-employment status | Yes/ no. | ✓ | ✓ | ✓ |
|  | Employment duration | Months, years. | ✓ | ✓ | ✓ |
|  | Employee contract status | Permanent/ fixed term / zero hours/ agency/ casual/freelance. | ✓ | ✓ | ✓ |
|  | Self-employed role | Sole trader/ owner/ freelancer/ other (FT). | ✓ | ✓ | ✓ |
|  | Job Responsibilities | 9 items: 1-5 scale: work patterns and location. | ✓ | ✓ | ✓ |
|  | Organisation size | 5 classes: 1 person/ micro (2-9)/ small (10-49)/ medium (50-249)/ large (≥250). | ✓ | ✓ | ✓ |
|  | Trade union membership (or similar) | Yes/No/Prefer not to state. | ✓ | ✓ | ✓ |
|  | Occupational Health presence at main workplace | Yes/No / don’t know/ not applicable. | ✓ | ✓ | ✓ |
|  | Use of work-related support | 8 classes: support and other (FT) in the last year. | ✓ | ✓ | ✓ |
|  |  |  |  |  |  |
|  | Job satisfaction [31] | 1 item: 1-5 scale to measure perceived job satisfaction. | ✓ | ✓ | ✓ |
|  | Job stressfulness [32] | 1 item: 1-5 scale to measure perceived job stress. | ✓ | ✓ | ✓ |
|  | Sickness absence | 1 item: 5 classes: number of sickness absence days in last year: No time/ <5 days/ 5-20 days/ >20 days/ not working. | ✓ | ✓ | ✓ |
|  | Turnover intentions [33] | 1 item: Yes/ no. | ✓ | ✓ | ✓ |
|  | Work Ability Index (WAI) [26] | 3 items: i) 0-10 rating: compared to lifetime best, current work ability; ii)1-5 rating: current work ability with respect to the physical demands of your work; iii) 1-5 rating: current work ability with respect to the mental demands of your work. | ✓ | ✓ | ✓ |
| **Work-related PROMs** |  |  |  |  |  |
|  | |  |  |  |  |
| Work productivity and activity impairment |  |  |  |  |  |
|  | Work Productivity and Activity Impairment Questionnaire: General Health V2.0 (WPAI:GH) [41] | Current employment status. | ✓ | ✓ | ✓ |
|  |  | In last 1/52: number of hours missed due to health problems. | ✓ | ✓ | ✓ |
|  |  | In last 1/52: number of hours missed not due to health problems. | ✓ | ✓ | ✓ |
|  |  | In last 1/52: number of hours worked. | ✓ | ✓ | ✓ |
|  |  | 1 item: rank 0-10: how much health problems affected work productivity, in last 1/52. | ✓ | ✓ | ✓ |
|  |  | 1 item: rank 0-10: how much health problems affected ability to perform regular daily activities, in last 1/52. | ✓ | ✓ | ✓ |
|  |  |  |  |  |  |
| Work self-efficacy |  |  |  |  |  |
|  | Work Self-Efficacy Scale (WSE-S) [28] | 3 items: rank 0-10: at present, confidence in: ability to work, manage condition at work, working not making condition worse. | ✓ | ✓ | ✓ |
|  |  | 3 items: rank 0-10: over the next year, motivation to work, importance of continuing work, confidence in continuing work. | ✓ | ✓ | ✓ |
|  |  |  |  |  |  |
|  | The Occupational Self-Efficacy Scale – Short Form (OSS-SF) [29] | 6 items: ranked strongly disagree to strongly agree (1-6) statements about coping with difficulties, problems, meeting goals and demands | ✓ | ✓ | ✓ |
|  |  |  |  |  |  |
| Social support at work |  |  |  |  |  |
|  | Demand Control Support Questionnaire (DCSQ) (Social Support at Work sub-scale) [30] | 6 items: 1-4 scale. Measuring perceived atmosphere at work, relationship with and perceived support from co-workers and superiors. | ✓ | ✓ | ✓ |
| iv. **Psychological and Health-Related Quality of Life (HRQoL) PROMs** | | |  |  |  |
| Depression | | |  |  |  |
|  | Patient Health Questionnaire (PHQ-2) [35] | 2 items: 0-3 scale. Self-administered anxiety screening tool. | ✓ | ✓ | ✓ |
| Anxiety |  |  |  |  |  |
|  | General Anxiety Disorder (GAD-7) [34] | 7 items: 0-3 scale. Self-administered depression screening. | ✓ | ✓ | ✓ |
| Health-related quality of life |  |  |  |  |  |
|  | EuroQol Five Dime[42]nsions questionnaire (EQ-5D-5L) [36] | 5-items: 0-5 scale (Mobility; Self-care; Usual activities; Pain/Discomfort; Anxiety/Depression; and health today rating 0–100 scale. | ✓ | ✓ | ✓ |
|  |  |  |  |  |  |
| v. **Health Resource Use** | |  |  |  |  |
| Use of NHS and Private Health Services | |  |  |  |  |
|  | Care from the GP Surgery | Number of visits in last 4/52.: GP/ practice Nurse/ OT/ physiotherapist, other (FT). | ✓ | ✓ | ✓ |
|  | Complementary Care | Number of visits in last 4/52: chiropractor/ osteopath/ other (FT). | ✓ | ✓ | ✓ |
|  | Use of NHS hospital outpatient clinic appointments for pain | Number of appointments in last 4/52. Department: rheumatology/ orthopaedics/ pain management clinic/ other (FT). | ✓ | ✓ | ✓ |
|  | Use of other NHS hospital clinic appointments for pain | Number of appointments in last 4/52: OT/ physiotherapist/ other (FT). | ✓ | ✓ | ✓ |
|  | Use of private (non-NHS) hospitals for pain | Number of days in last 4/52. | ✓ | ✓ | ✓ |
|  | Use of private (non-NHS) healthcare professionals for pain | Number of visits in the last 4/52: doctor/ OT / physiotherapist/ chiropractor/ osteopath/ other (FT). | ✓ | ✓ | ✓ |
|  | Pain medication | Name and number of days in last 4/52: Prescribed/ complementary/ over the counter. | ✓ | ✓ | ✓ |
|  |  |  |  |  |  |
| vi. **Technology-adoption PROMs (intervention only)** | |  |  |  |  |
| Pain-at-Work Toolkit Intervention |  |  |  |  |  |
|  | Technology Acceptance Questionnaire (TAM) [19] | 8 items: 1-5 scale: Whether the Pain-at-Work Toolkit increased knowledge and awareness, was relevant, easy to use, clear and understandable, changed attitudes, motivation, or help-seeking behaviours to pain management at work. |  | ✓ |  |
|  |  | Identify any barriers to using the Pain-at-Work Toolkit (FT). |  | ✓ |  |
|  |  | Whether using the Pain-at-Work Toolkit led to behaviour change (FT). |  | ✓ |  |
|  |  | Whether the Pain-at-Work Toolkit was useful and whether they would recommend it to others. |  | ✓ |  |
| Text message reminders |  |  |  |  |  |
|  |  | Whether text reminders were acceptable, and whether they prompted access to the Pain-at-Work Toolkit or the OT support. |  | ✓ |  |
|  |  |  |  |  |  |
|  |  |  |  |  |  |
| Pain-at-Work Toolkit usage | Google analytics | To collect data on fidelity (link distribution and receipt), frequency and intensity of engagement (initial access, page, and section views), duration of engagement (time spent using the Toolkit), type of engagement (active or passive). |  |  | ✓ |
| **WP2** Health care resources use | Health Economic Questionnaire [37] | Items on SAU costs, healthcare resource use costs, medicines use, primary care consultations, hospital stays and outpatient visits for reasons related to chronic pain/anxiety/depression, and quality of life measurement. | ✓ | ✓ | ✓ |
| **WP3 Evaluation and Implementation** | Interviews (intervention participants) | Usage and perceptions of the intervention determined at T2. |  |  | ✓ |
|  | Participant feedback form | Usage and perceptions of the intervention determined at T2. |  |  | ✓ |
|  | Interviews (stakeholders) | Qualitative interviews will explore whether stakeholders and employers find the intervention acceptable |  |  | ✓ |

Abbreviations: BMI – Body Mass Index; FT – free text option; GP – General Practitioner; kg- kilograms; m – metres; PROMS – Participant Reported Outcome Measures; T0 – Timepoint (baseline); T1 – Timepoint 1 (3 months); T2 – Timepoint 2 (6 months); SAU – Support-as- usual
